# Insecticide-treated net wall hangings for malaria vector control: an experimental hut study in north-eastern Tanzania

**DOI:** 10.1186/1475-2875-13-366

**Published:** 2014-09-17

**Authors:** Corine Ngufor, Patrick Tungu, Robert Malima, Matthew Kirby, William Kisinza, Mark Rowland

**Affiliations:** London School of Hygiene and Tropical Medicine (LSHTM), London, WC1E 7HT UK; National Institute of Medical Research (NIMR), Muheza, Tanzania

**Keywords:** Insecticide-treated net wall hangings, Pirimiphos methyl, Experimental huts, Muheza, Kdr, Insecticide resistance

## Abstract

**Background:**

Alternative long-lasting, practical and effective tools for applying insecticides on home walls against malaria vectors need to be developed. The use of wall hangings made from netting on interior walls for aesthetic purposes is a common practice in rural communities. Insecticide-treated net wall hangings can be produced in a long-lasting format and used in an approach that simulates indoor residual spraying (IRS).

**Methods:**

The efficacy of net wall hangings (NWH) treated with the residual organophosphate insecticide, pirimiphos methyl (1 g/sq m), was evaluated in experimental huts against malaria vectors in Muheza, Tanzania. To determine the optimum level of wall coverage required, NWH were tested on ceiling only, two walls, four walls, or four walls plus ceiling. Comparison was made with deltamethrin-treated NWH on two walls.

**Results:**

Pirimiphos methyl (p-methyl)-treated NWH (on two walls) killed significantly higher proportions of anophelines (92% of *Anopheles gambiae* and 79% of *Anopheles funestus*) than the deltamethrin-treated NWH (15% of *An. gambiae* and 17% of *An. funestus*) (P < 0.001). WHO susceptibility tests showed that the local vector population was susceptible to the organophosphates but resistant to pyrethroids. Mortality rates were significantly higher in huts with p-methyl NWH on two walls (92% for *An. gambiae* and 79% for *An. funestus*) than on ceiling only (61% for *An. gambiae* and 62% for *An. funestus*, P < 0.05). There was no improvement in mortality when wall coverage with p-methyl NWH increased beyond two walls. Blood-feeding rates with p-methyl NWH were generally high across all the treatments (52-77%) and did not differ significantly from the control (64-67%). There was no evidence of reduced blood-feeding or increased exiting with increase in wall coverage with p-methyl NWH.

**Conclusions:**

Net wall hangings are an effective means of delivering insecticides in the domestic environment against malaria vectors. They could be more practical and acceptable than IRS thus showing enormous potential for malaria vector control. Appropriate binding or incorporation technology needs to be developed to enable the production of p-methyl NWH with residual activity lasting over a number of years.

## Background

Indoor residual spraying (IRS) has a distinguished historical role in the control of malaria. It has been one of the main interventions leading to the elimination of malaria in half of the world’s regions, such as in much of Southern Europe, North America, Japan, Central and South Asia and Latin America [1, 2]. In recent years, IRS has been scaled up significantly in Africa, contributing to the recent reductions in malaria morbidity and mortality [3, 4]. However, sustaining user compliance and overcoming the operational challenges associated with the implementation of IRS remains a major challenge [5] especially in holo-endemic areas in sub-Saharan Africa.

Insecticide-treated materials can be applied on home walls in a novel approach that simulates IRS. Long-lasting pyrethroid-treated plastic sheeting, which was originally developed for malaria control in refugee situations [6], has also been produced for use on the interior of home walls [7, 8]. This tool is popularly referred to as durable lining (DL). Pyrethroid-treated DL is manufactured using binding technology, which allows the insecticide to diffuse slowly to the surface in a controlled fashion, making it a long-lasting alternative to IRS. In a recent multicentre study, pyrethroid DL showed potential to overcome user-fatigue and the operational challenges associated with the use of recurrent IRS treatments in holo-endemic areas [8]. However, there are some concerns over the time required to install DL in homes and the durability of the plastic sheeting on home walls. More practical and flexible versions of this approach need to be developed.

The use of hangings made from different sorts of materials on interior home walls for the purpose of decoration is a common human practice. Home-owners in rural Africa sometimes cover their walls with wall hangings made from netting or curtain material. Insecticide-treated net wall hangings could operate in a similar manner to IRS if mosquitoes that enter the home rest on them. Because the netting material is widely available and much lighter in weight than plastic sheeting, net wall hangings (NWH) could be a more practical and acceptable means of delivering insecticides in the domestic environment.

Pyrethroids remain the most suitable insecticides for treating long-lasting, insecticide-treated bed nets (LLINs) owing to their rapid knockdown effect, low cost and low mammalian toxicity. To reduce selection pressure for pyrethroid resistance and help preserve this class of insecticides, the WHO recommends that pyrethroids be reserved for LLINs since LLINs will remain the most important public health intervention [9, 10]. Hence non-pyrethroid versions of DL and NWH are more desirable. They could be used on their own or in combination with LLINs for improved control of pyrethroid-resistant malaria vectors and for managing insecticide resistance. The current study investigated the efficacy of NWH treated with pirimiphos methyl (p-methyl) CS (Actellic®300 CS), a WHO approved organophosphate insecticide, in experimental huts in Muheza, northeastern Tanzania. Comparison was made to pyrethroid (deltamethrin)-treated NWH. To determine the level of wall coverage required for optimum impact, NWH were tested on ceiling only, two walls, four walls, or four walls plus ceiling. WHO susceptibility tests were performed to investigate the existence of resistance to a range of insecticides recommended for IRS.

## Methods

### Study sites and experimental huts

The study was carried out in six experimental huts of East African design in Zeneti village in Muheza District, northern Tanzania (5^0^13′S and 38^0^39′E, altitude 193 m). *Anopheles gambiae s.l*. is the predominant vector in the wet season while *Anopheles funestus* is predominant in the dry season [11]. The trial ran between June and July of 2011 during the months that both species co-exist. The experimental huts conformed to the WHOPES-approved design [12] with some minor adjustments as described by Malima *et al.* [13]. The huts are made of brick plastered with cement on the inside with a corrugated iron roof, which is lined with palm thatch and has an eave gap below. There are veranda and window traps on each side of the hut. Two of the verandas were left open to allow mosquitoes to enter the huts through the eaves while the other two were screened to capture any mosquitoes that exited via the eaves.

### Treatment and hanging of net wall hangings

Netting material used was 100-denier polyester netted fabric purchased from the local market. These were treated by dipping in either pirimiphos methyl CS (Actellic® 300CS, Syngenta, Basle, Switzerland) at 1 g/sq m or deltamethrin SC (K-Othrine 10SC, Bayer, Monnheim, Germany) at 55 mg/sq m. Treated NWH were left to dry in the shade for 24 hours before being hung onto the hut walls. In order to avoid contamination of the walls when rotating the treatments between the huts, an underlay of untreated plastic sheeting was used to separate the walls from the treated materials and these were rotated along with the respective treatments. Treated nettings were simply hung onto nails that had been fitted at the top edge of the hut walls. Areas of the treated NWH covering the windows were then cut out to allow exit of mosquitoes to window traps.

### Sleepers and treatments

Six adult men served as volunteer sleepers and were rotated between huts on successive nights to adjust for any variation in individual attractiveness to mosquitoes. Sleepers gave informed consent and were provided with chemoprophylaxis prior to the trial. They slept in the huts from 20:00 to 05:00 each night. White sheets were laid over the veranda and room floors to ease the collections of knocked down mosquitoes. Mosquitoes were collected each morning at 05:00 from under bed nets, floors, walls, ceilings, verandas, and window traps using aspirators and torches. The collections were transported to the laboratory where mosquitoes were identified to species and scored as blood fed or unfed and live or dead. Live mosquitoes were held in netted plastic cups and supplied with 10% glucose solution and delayed mortality was recorded after 24 hours. Male mosquitoes were not scored. Data were collected for 36 nights.

Ethical approval for the study was obtained from the Ethics Review Boards of the London School of Hygiene and Tropical Medicine and the Tanzanian National Institute of Medical Research.

The following six treatments were compared in the experimental huts:

1. Untreated control hut
2. Deltamethrin NWH on two walls
3. P-methyl NWH on ceiling
4. P-methyl NWH on two walls
5. P-methyl NWH on four walls
6. P-methyl NWH on four walls and ceiling (full coverage)

The treatments were rotated through the huts on a weekly basis following a Latin Square design to account for positional differences in attractiveness between the huts.

### Entomological outcomes

The impact of each treatment was expressed in terms of the following entomological outcomes:

1. Deterrence: percentage reduction in the number of mosquitoes caught in treated hut relative to the number caught in the control hut
2. Exiting rates: due to potential irritant effect of treatments expressed as percentage of the mosquitoes collected from the veranda trap.
3. Blood feeding rates: percentage of blood fed mosquitoes collected from the experimental huts.
4. Blood feeding inhibition which is the reduction in blood feeding rate relative to the control. Blood feeding inhibition (%) was calculated as follows:

Where Bfu is the proportion of blood-fed mosquitoes in the untreated control huts and Bft is the proportion of blood-fed mosquitoes in the huts with a specific insecticide treatment.

5. Mortality rates: percentage of dead mosquitoes in hut at the time of collection and after a 24-hour holding period.

### Residual activity

To determine the residual activity of the treated NWH, WHO cone bioassays were performed *in situ* at the beginning and the end of the trial. A total of 100 mosquitoes of the laboratory susceptible *An. gambiae* Kisumu strain were tested on each type of treated NWH in the experimental huts. The mosquitoes were exposed for 30 minutes following WHOPES guidelines [12]. Mortality was recorded after a 24-hour holding period.

### Susceptibility testing

To test for the existence of resistance to a range of insecticides recommended for IRS, WHO susceptibility tests were performed on adult *An. gambiae* mosquitoes, which emerged from larvae collected from the study area. Mosquitoes at three to five days old were exposed for one hour to filter papers treated to the recommended diagnostic dose of each insecticide in cylinder bioassays [14]. For pirimiphos methyl, a range of concentrations (0.025-0.25%) was tested and comparison was made to the laboratory-susceptible *An. gambiae* Kisumu strain. For each insecticide and each concentration of p-methyl, a total of 95–100 adult female mosquitoes were tested and the proportion dead recorded after 24 hours.

### Knock down resistance (*kdr*) genotype testing

To investigate the presence of the *kdr* (L1014S) gene in the *An. gambiae* vector population in Muheza, genomic DNA was extracted from a random sample of mosquitoes collected from the experimental huts using the Livak procedure [15]. Molecular detection of the L1014S mutation alleles was carried out by real-time Taqman PCR as described by Bass *et al.* [16].

### Data analysis

The numbers of mosquitoes entering the huts with the different treatments was analysed by negative binomial regression. The effects of the treatments on each of the main proportional entomological outcomes (exophily, blood feeding and mortality) were assessed using binomial generalized linear mixed models (GLMMs) with a logit link function, fitted using the ‘lme4’ package for R. A separate model was fitted for each outcome and for each mosquito species. In addition to the fixed effect of each treatment, each model included random effects to account for the following sources of variation: between the six huts used in the studies; between the six sleepers who slept in the huts; between the six weeks of the trial; and finally an observation-level random effect to account for variation not explained by the other terms in the model (over dispersion).

## Results

### Susceptibility tests

**Figure 1:**
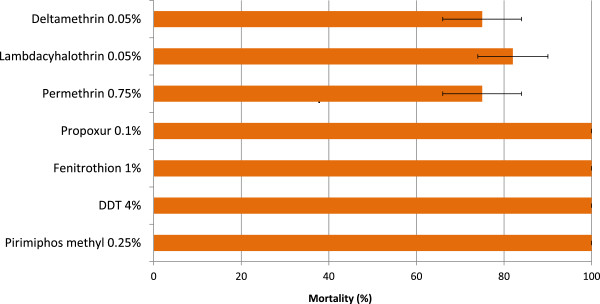
**Susceptibility of** ***Anopheles gambiae*** **from Muheza, Tanzania to insecticides.** Mortality (%) in WHO cylinder bioassays during hut trials. Error bars represents 95% confidence intervals.

The WHO susceptibility tests showed that the local vector population was resistant to pyrethroids but susceptible to organophosphates (Figure 1). Mortality rates of wild anopheline mosquitoes from the study site were 100% across all the concentrations of p-methyl tested, confirming susceptibility to the organophosphate. The genotyping results revealed a *kdr* allele (L1014S) frequency of 0.22 in a random sample of 47 *An. gambiae s.l.* mosquitoes collected from the experimental huts.

### Experimental hut trial

**Figure 2:**
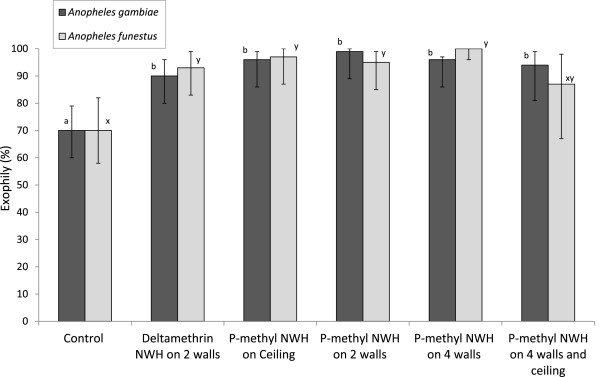
**Exiting rates of anopheline mosquitoes in experimental huts with insecticide-treated net wall hangings.** For each species, values for bars bearing the same letter label are not significantly different at 5% level. Error bars represents 95% confidence intervals.

**Figure 3:**
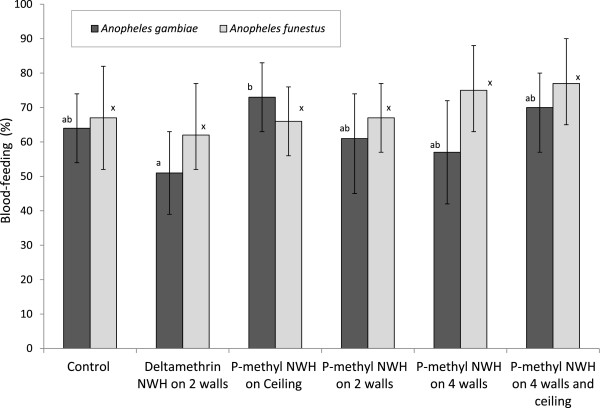
**Blood-feeding rates of anopheline mosquitoes in experimental huts with insecticide-treated net wall hangings.** For each species, values for bars bearing the same letter label are not significantly different at 5% level. Error bars represents 95% confidence intervals.

**Figure 4:**
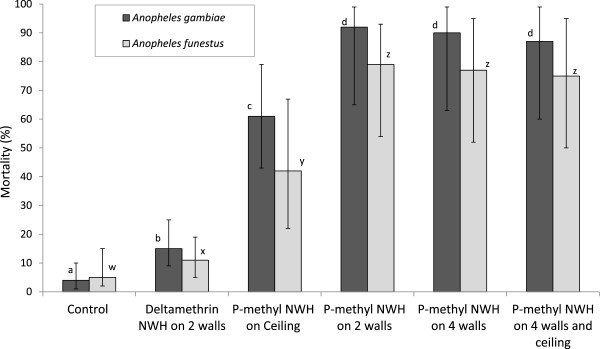
**Mortality of anopheline mosquitoes in experimental huts with insecticide-treated net wall hangings.** For each species, values for bars bearing the same letter label are not significantly different at the 5% level. Error bars represents 95% confidence intervals.

The numbers of wild anopheline mosquitoes entering, feeding and dying in the experimental huts during the trial are presented in Table 1 for *An. gambiae* and Table 2 for *An. funestus*. The exiting, blood-feeding and mortality rates are presented in Figures 2, 3 and 4 respectively. A total of 423 *An. gambiae* and 277 *An. funestus* were collected from the experimental huts during the trial (Tables 1 and 2). The highest numbers were collected in the control hut. For both species, the average catch per night did not differ significantly between the p-methyl NWH on two walls and the pyrethroid DL on two walls. The level of deterrence with p-methyl NWH showed an increase with increasing wall coverage.

**Table 1:** **Numbers of** ***An. gambiae*** **entering, feeding and dying in experimental huts with insecticide treated NWH**

Hut treatment	Control (untreated DL)	Deltamethrin NWH on 2 walls	P-methyl NWH on Ceiling	P-methyl NWH on 2 walls	P-methyl NWH on 4walls	P-methyl NWH on 4 walls and ceiling
Total females caught	171	86	57	60	35	14
Average catch per night	4.8^a^	2.4^b^	1.6^b^	1.7^b^	1.0^bc^	0.4^c^
Deterrence (%)	0	50	67	65	80	92
Total females blood fed	109	44	42	37	20	10
Blood feeding Inhibition (%)	0	20	0	5	11	0
Total dead	7	13	35	55	31	12
Corrected mortality (%)	0	11	59	92	90	86

Values along a row sharing the same letter superscript are not significantly different at the 5% level.

**Table 2:** **Numbers of** ***An. funestus*** **entering, feeding and dying in experimental huts with insecticide treated NWH**

Hut Treatment	Control (untreated DL)	Deltamethrin NWH on 2 walls	P-methyl NWH on ceiling	P-methyl NWH on 2 walls	P-methyl NWH on 4walls	P-methyl NWH on 4 walls and ceiling
Total females caught	136	60	28	31	15	7
Average catch per night	3.8^a^	1.7^b^	0.8^c^	0.9^bc^	0.4^c^	0.2^c^
Deterrence (%)	0	56	79	77	89	95
Total females blood fed	91	37	19	21	11	5
Blood feeding inhibition (%)	0	1	0	0	0	0
Total dead	7	7	12	25	11	5
Corrected mortality (%)	0	6	39	78	76	74

Values along a row sharing the same letter superscript are not significantly different at the 5% level.

### Hut exiting rates

Exiting rates were significantly higher in the huts with the treated NWH than the control (Figure 2) (for both species, P < 0.05 for each treatment relative to control). The proportion exiting did not differ between the deltamethrin NWH (two walls) hut and the p-methyl NWH (two walls) hut for either species (P = 0.71 for *An. gambiae*, P = 0.85 for *An. funestus*). There was no evidence of a relationship between treatment-induced exiting and the level of wall coverage with p-methyl NWH.

### Blood feeding

Blood-feeding rates were very high across all the treatments (Figure 3) hence the treated NWH provided very little or no blood-feeding inhibition relative to the control (Tables 1 and 2). The proportions blood-fed in huts with p-methyl NWH for both anopheline species (range of 52-75%) were not significantly different from the control hut (64% of *An. gambiae* and 67% of *An. funestus*, P > 0.05) (Figure 3). The proportion feeding in the hut with deltamethrin NWH on two walls (51% of *An. gambiae* and 61% of *An. funestus*) was also not significantly different from the proportion feeding in the hut with p-methyl NWH on two walls (62% of *An. gambiae* and 67% of *An. funestus*) (P = 0.07 for *An. gambiae*, P = 0.1 for *An. funestus*). As with exophily, the data showed no evidence of a relationship between the level of wall coverage with p-methyl NWH and blood-feeding rate by *Anopheles* species.

### Mortality

Figure 4 presents the mortality rates in the different experimental huts. The treated NWH generally killed significantly larger proportions of mosquitoes than the control. Mortality of both anopheline species was much higher with p-methyl NWH on two walls (92% of *An. gambiae* and 77% of *An. funestus*) compared to deltamethrin NWH on two walls (15% of *An. gambiae* and 17% of *An. funestus*) (P < 0.001 for both species) (Figure 4). The proportion dead also increased significantly in the hut with p-methyl NWH on two walls (92% for *An. gambiae* and 79% for *An. funestus*) compared to the huts with p-methyl NWH on ceiling only (61% for *An. gambiae* and 62% for *An. funestus*) (P = 0.004 for *An. gambiae* and P = 0.01 for *An. funestus*). Mortality rates in huts with p-methyl NWH on four walls and four walls plus ceiling were 87% and 90% respectively for *An. gambiae* and 75% and 77% respectively for *An. funestus* but these values did not differ significantly from that with p-methyl NWH on two walls (P > 0.05 for both species) (Figure 4). Hence, the results did not show an improvement in mortality of either species when wall coverage with p-methyl NWH increased beyond two walls.

### Residual activity

**Figure 5:**
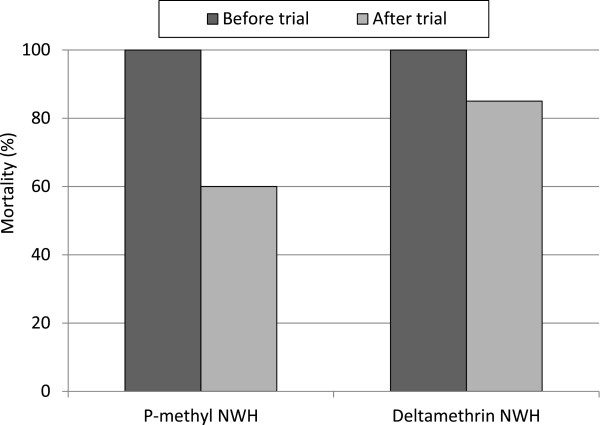
**Mortality (%) of laboratory susceptible** ***Anopheles gambiae*** **Kisumu exposed to treated net wall hangings in cone bioassays before and after the experimental hut trial.**

At the beginning of the trial both deltamethrin and p-methyl-treated NWH induced 100% mortality with the laboratory-susceptible *An. gambiae* Kisumu strain in WHO cone bioassays. By the end of the trial (after six weeks), mortality with p-methyl-treated NWH declined to 60% but remained >80% with deltamethrin-treated NWH (P < 0.01) (Figure 5). Deltamethrin (at 55 mg/sq m) therefore showed a longer residual activity on the nylon netting material than p-methyl (at 1 g/sq m). No mortality was recorded in the control hut.

## Discussion

New or improved practical and adaptable tools for delivering insecticides against malaria vectors are urgently needed. The current study was designed to investigate the potential of insecticide-treated NWH as a novel system for delivering insecticides indoors. The results indicate that mosquitoes will readily rest on them and be killed in the process.

P-methyl-treated NWH (on two walls) induced much higher mortality rates than deltamethrin-treated NWH (on two walls). The vector population was susceptible to organophosphates but resistant to pyrethroids as demonstrated in the WHO susceptibility bioassays. Insecticide resistance could have combined with pyrethroid excitorepellency to reduce the overall level of mortality in the partially treated rooms by causing the re-distribution of the surviving resistant mosquitoes on the untreated walls where they settle and evade any toxic effect of the insecticide. A previous survey carried out in 2009/2010 in the study area (Muheza) showed full susceptibility to pyrethroids [17]. The present study therefore demonstrates a rapid development of resistance in this vector population between 2010 and 2011 and the impact that this may have on pyrethroid-based vector control tools. This rapid change from susceptibility to resistance could be due to high selection pressure posed by the massive distribution of LLINs in the Muheza district in 2010 following the Tanzanian government’s catch-up campaign to distribute nine million LLINs to children aged less than five years [18, 19]. Though the *kdr* gene was detected, further studies need to be performed to investigate the presence of other mechanisms of resistance to pyrethroids, which, in addition to the *kdr*, may have contributed to the level resistance observed. The impact of this shift in resistance status on the efficacy of the LLINs being used in the area also needs to be assessed.

Increasing wall coverage with p-methyl NWH from two walls to four walls or four walls plus ceiling (full coverage) did not improve on mortality. In contrast, a previous study with pyrethroid DL in an area with much higher levels pyrethroid resistance showed that it was necessary to cover all four walls before a significant level of mortality could be achieved [7]. However, people do not always cover all their walls with wall hangings since it may be aesthetically more appealing and more practical to cover a few walls. The results of the current study therefore suggest that p-methyl NWH could be a more scalable and cost-effective intervention than pyrethroid-treated NWH or pyrethroid DL. Nevertheless, the vector population was fully susceptible to the organophosphate but resistant to the pyrethroid. The performance of p-methyl NWH and the level of wall coverage required may depend on the resistance status of the targeted vector population.

Although mortality rates were high, blood-feeding rates with the treated NWH were generally high. This provides evidence that NWH act like IRS rather than insecticide-treated nets. With IRS-like treatments mosquitoes would normally first feed on the person sleeping in a hut or house before resting on the IRS-treated wall where they pick up the insecticide, unless there is an additional tool to prevent blood feeding. In a parallel study, combining p-methyl NWH with LLINs improved blood-feeding inhibition significantly (due to the LLIN component) [20]. Such combinations have also shown potential for insecticide resistance management whereby insect genotypes which are resistant to the insecticide in one intervention can be killed by the other insecticide if they are not resistant to both insecticides [21–23].

Although blood feeding rates with p-methyl NWH were high, mosquitoes were deterred from entering the treated huts compared to the control hut and this deterrent effect increased with increasing wall coverage with p-methyl NWH. Previous studies also demonstrated an increase in hut deterrence as wall coverage with pyrethroid DL increased [7]. Deterrence of mosquitoes from insecticide treated experimental huts is usually induced by the irritant or repellent effect of the insecticide. While this effect has been mostly associated with pyrethroids, some studies have also shown reduced mosquito entry in experimental huts treated with microencapsulated p-methyl IRS [24, 25]. By deterring mosquitoes from treated homes, p-methyl NWH shows potential to significantly reduce human-vector contact which could contribute substantially to reducing malaria transmission.

P-methyl showed a shorter residual activity on NWH than deltamethrin. Studies with this slow-release microencapsulated formulation of the insecticide have shown prolonged residual activity on cement walls [24]. The insecticide particles probably scaled off the treated netting material over time due to movements during the rotations. Nevertheless, because the study was designed as a proof of concept to demonstrate the relevance of NWH, the nettings used were hand-dipped, so the short residual activity is not unexpected. The netting material is a very benign substrate and as observed with ITNs, many kinds of insecticides can be readily applied to netting. They can then be delivered on walls through this NWH approach. The mortality rates observed in the current study show that p-methyl-treated NWH have potential to control indoor resting malaria vectors.

It took less than 10 minutes for a team of two individuals to set up NWH on the four walls of an experimental hut whereas a previous study reported 60–75 minutes for three individuals to install pyrethroid DL in a house [8]. NWH is also lighter in weight and can be simply hung onto nails fitted at the edges of the ceiling by home-owners. The DL plastic sheeting on the other hand is heavier and its installation usually requires a skilled team of individuals to ensure that it is well fitted as to reduce the risk of it falling off the wall. Hence NWH may be more practical or popular than DL or IRS. However, to guarantee added benefit from wide scale use of p-methyl NWH over standard IRS, the residual activity will need to last for years rather than months. Advanced binding technology therefore needs to be applied to develop long-lasting versions of NWH. In the meantime, hand-treated NWH can be used in the place of IRS in transitory house structures and in houses with mud walls, which usually show very low residual activity with IRS applications [24]. NWH could also be used to cover eave gaps and cracks and crevices on walls as to reduce mosquito entry into homes.

### Study limitations

The numbers of mosquitoes collected in some of the huts were few especially the huts with p-methyl NWH on four walls and four wall plus ceiling. However, this effect could be attributed to the low density of mosquitoes in the study area and increased deterrence of mosquitoes from these huts posed by the higher levels of wall coverage with p-methyl NWH. Nevertheless, the trends observed were clear showing significantly higher mortality rates in huts with p-methyl NWH than huts with the untreated control and the pyrethroid NWH.

## Conclusion

The results demonstrate that NWH are an effective means of delivering insecticides in the domestic environment since mosquitoes rested on them and were killed in the process. They could be more practical and acceptable than IRS or DL showing potential for malaria vector control. Appropriate binding or incorporation technology needs to be developed to enable the production of p-methyl NWH with residual activity lasting over a number of years.
